# Factors that account for inter-individual variability of lateralization performance revealed by correlations of performance among multiple psychoacoustical tasks

**DOI:** 10.3389/fnins.2014.00027

**Published:** 2014-02-13

**Authors:** Atsushi Ochi, Tatsuya Yamasoba, Shigeto Furukawa

**Affiliations:** ^1^Human Information Science Laboratory, NTT Communication Science Laboratories, NTT CorporationAtsugi, Japan; ^2^Department of Otolaryngology, Faculty of Medicine, University of TokyoTokyo, Japan

**Keywords:** interaural time difference, interaural level difference, level discrimination, correlation, temporal fine structure, phase locking

## Abstract

This study explored the source of inter-listener variability in the performance of lateralization tasks based on interaural time or level differences (ITDs or ILDs) by examining correlation of performance between pairs of multiple psychoacoustical tasks. The *ITD*, *ILD*, *Time*, and *Level* tasks were intended to measure sensitivities to ITD; ILD; temporal fine structure or envelope of the stimulus encoded by the neural phase locking; and stimulus level, respectively. Stimuli in low- and high-frequency regions were tested. The low-frequency stimulus was a harmonic complex (*F*_0_ = 100 Hz) that was spectrally shaped for the frequency region around the 11th harmonic. The high frequency stimulus was a “transposed stimulus,” which was a 4-kHz tone amplitude-modulated with a half-wave rectified 125-Hz sinusoid. The task procedures were essentially the same between the low- and high-frequency stimuli. Generally, the thresholds for pairs of ITD and ILD tasks, across cues or frequencies, exhibited significant positive correlations, suggesting a common mechanism across cues and frequencies underlying the lateralization tasks. For the high frequency stimulus, there was a significant positive correlation of performance between the ITD and Time tasks. A significant positive correlation was found also in the pair of ILD and Level tasks for the low- frequency stimulus. These results indicate that the inter-listener variability of ITD and ILD sensitivities could be accounted for partially by the variability of monaural efficiency of neural phase locking and intensity coding, respectively, depending of frequency.

## Introduction

Performance in lateralization tasks based on interaural time and level differences (ITDs or ILDs), the major cues for horizontal sound localization, often varies markedly among listeners. Lateralization behavior is a product of multiple stages of auditory processing, and thus the listener's performance should reflect the efficiencies of the individual processes by varying degrees. We consider that the processing of the ITD or the ILD in the auditory system consist of two or more stages. The earliest is the peripheral stage, in which the auditory information is processed individually for different ears. At this stage, the temporal structure and intensity of sounds at each ear are encoded to neural signals in the form of the timing and number of auditory nerve firings. The outputs of this stage of processing are fed to processes at the binaural interaction stage, where the relative timing and number of neural firings for the two ears are compared. This binaural interaction stage is followed by the subsequent higher-order processes.

The present study aimed to evaluate the relative contributions of these processing stages to the inter-listener variabilities in lateralization performance. We measured listeners' monaural sensitivities to the temporal structure and intensity of a sound stimulus, as well as their ITD and ILD sensitivities. The hypothesis was that the lateralization performance based on ITD is predominantly determined by the efficiency of temporal structure coding by neural phase-locking at the peripheral processing stage. If this is true, we would expect that the ITD-based lateralization performance correlates with the performance of a non-lateralization task, which reflects sensitivity to the temporal structure of the stimulus that is presumed to be represented by phase locking. A similar hypothesis and prediction are possible in terms of the relationship between ILD-based lateralization and peripheral intensity coding.

The authors are not aware of a study examining the extent to which monaural intensity (or level) encoding efficiency could account for individual differences in ILD sensitivity. On the other hand, the above hypothesis on the relationship between temporal structure coding and ITD sensitivity is supported by studies on the effects of aging and/or hearing-impairment. Groups of aged listeners (Strouse et al., 1998; Hopkins and Moore, 2011) with sensorineural hearing impairment (Strelcyk and Dau, 2009; Hopkins and Moore, 2011) and those with auditory neuropathy (Zeng et al., 2005) exhibited degraded performance more or less specific to the ITD-based lateralization task and to tasks that measure monaural sensitivity to temporal structure, in comparison to control groups. Within-listener correlation between the two types of tasks has also been reported. Strelcyk and Dau (2009) found a positive correlation between the FM detection threshold (considered to be indicative of sensitivity to the temporal fine structure, TFS) and ITD-based lateralization threshold for hearing-impaired listeners (there was no report for normal-hearing listeners). A similar relationship between the monaural sensitivity to the TFS and the binaural sensitivity to interaural phase differences was also reported for a pooled population of young and aged listeners with and without hearing impairment (Hopkins and Moore, 2011). Nevertheless, it is uncertain whether the positive correlation could be applicable also to the population of normal-hearing listeners. A possibility is that a long-term impairment of a single mechanism (i.e., peripheral TFS coding) affects the efficiency of another independent mechanism (i.e., central binaural processing), leading to an apparent correlation of performance. Strouse et al. (1998) found a strong positive correlation between the monaural temporal-gap detection threshold and ITD discrimination threshold for a group of normal-hearing young listeners, although such a positive correlation was not found for aged listeners. It should be noted, however, that the gap detection task is considered to focus on the sensitivity to the temporal envelope, rather than on that to the cycle-by-cycle TFS of the stimulus.

A secondary aim of the present study was to examine whether mechanisms for processing the ITD (and ILD) are essentially the same across operating frequency regions. It has been argued that essentially the same binaural mechanism is involved in processing ITDs at low and high frequencies, and apparent differences in ITD sensitivities between the frequency regions reflect differences in input to the system (Van De Par and Kohlrausch, 1997; Bernstein, 2001): When high-frequency “transposed stimulus” (see Material and Methods) is used so that the pattern of neural phase locking to the envelope of the stimulus resembles that to TFS of a low-frequency stimulus, listeners' performance for ITD-related tasks should be comparable. Furukawa (2008), however, found that the degree of ITD and ILD cue interaction in lateralization tasks was smaller for low- than for high-frequency regions, even when the inputs to the binaural system were made comparable by using low-frequency tones and high-frequency transposed stimuli. This implies that a more-or-less independent ITD processor exists in the low frequency region, whereas in the high-frequency region, ITD is processed by a mechanism that is common for ILD processing. In this study, we used low- and high-frequency stimuli and examined the relationship between the lateralization tasks and the monaural temporal/intensity-related tasks for each type of the stimuli. Qualitatively different results between the stimulus types would imply the involvement of separate binaural mechanisms in lateralization depending on stimulus frequency.

## Materials and methods

### Listeners and apparatus

Twenty-two adults (10 males and 12 females; 19–43 years old, mean 32.0) participated in the experiment as listeners. All gave written informed consent, which was approved by the Ethics Committee of NTT Communication Science Laboratories. The listeners showed normal audiometric thresholds (<25 dB HL) at frequencies of 250, 500, 1000, 2000, 4000, and 8000 Hz. They had no symptoms of hearing loss and had never been diagnosed as having hearing loss by medical examination. All testing took place in a double-walled sound booth. The listener was seated in front of a computer monitor, which displayed indicators for observation intervals of the forced-choice task and buttons for responses (described later).

Stimuli were digitally synthesized by a personal computer (sampling frequency: 44.1 kHz) and generated by using a digital-to-analog converter with a resolution of 24 bits (M-AUDIO, Transit USB). The signals were amplified and presented to the listener through Sennheiser HDA200 headphones.

MATLAB (Mathworks, Inc.) software was used for stimulus synthesis, experimental control, and data analyses.

### Stimuli

The low- and high-frequency stimuli were designed to assess the listener's ability to use information based on neural phase-locking to the stimulus TFS and envelope, respectively, in the ITD and Time tasks. Essentially the same stimuli were used also in the ILD and Level tasks (See section Procedures for the descriptions of the four tasks).

The low-frequency stimulus was a spectrally shaped multicomponent complex (SSMC), which was a harmonic complex with a fundamental frequency (*F*_0_) of 100 Hz, consisting of the 7th to 14th harmonics. The components were added in the sine phase. We adopted stimulus parameters as in Moore and Moore (2003) to prevent the listener from using spectral cues (or the excitation-pattern cues) when conducting the tasks: The spectral envelope had a flat passband and sloping edges (5 × *F*_0_ centered at 1100 Hz).The overall level of the complex was 54 dB SPL. Threshold equalizing noise (TEN, Moore et al., 2000), extending from 125 to 15000 Hz, was added to mask combination tones and help ensure that the audible parts of the excitation patterns evoked by the harmonic and frequency-shifted tones were the same in the Time task (described later). The TEN level at 1 kHz was set at 30 dB/ERBN, which was 15 dB below the level of the 1100-Hz component.

The high-frequency stimulus was a “transposed stimulus,” which was a 4-kHz tone carrier amplitude-modulated with a half-wave rectified 125-Hz sinusoid. It is considered that the auditory-nerve firing is phase locked to the modulator waveform, which provides the cue for judging the ITD and modulation rate of the stimulus (Van De Par and Kohlrausch, 1997; Bernstein, 2001). For the present stimulus, the modulation frequency of 125 Hz was chosen because that was the frequency with which human listeners exhibited the highest ITD sensitivity in the study by Bernstein and Trahiotis (2002).The overall level of the transposed stimulus was set to 65 dB SPL. A continuous, low-pass filtered Gaussian noise (cutoff frequency 1300 Hz; spectrum level 20 dB SPL) was added to prevent the listener from using any information at low spectral frequencies (e.g., combination tones).

### Procedures

#### General procedure

A two-interval two-alternative forced-choice (2I-2AFC) method was used to measure the listener's sensitivities to stimulus parameters. The listener was instructed to choose the “signal” interval by mouse-clicking one of two buttons displayed on a computer monitor or by pressing a corresponding key on a keyboard. Feedback was given to indicate the correct answer after each response. The two-down/one-up adaptive tracking method was used to estimate discrimination thresholds, corresponding to 70.7% correct (Levitt, 1970). One session of adaptive tracking lasted until twelve turnpoints were obtained. The first two sessions of each task and stimulus type were performed as practice sessions. When the tracking results appeared unstable for a listener with a task, two or three additional practice sessions were added for the listener/task/stimulus. A total of 8–10 sessions besides the practice sessions were conducted for each listener/task/stimulus. The thresholds were computed as the average of all the non-practice sessions. One session set consisted of two consecutive sessions for one task/stimulus. The order of session sets for tasks and stimuli were randomized for each subject in order to reduce the influence of the training and/or order effect.

#### Task specific procedures

***ITD task***. In a 2I-2AFC trial, stimuli in the two intervals had ITDs of +ΔITD/2 and −ΔITD/2 μs, respectively (positive and negative ITDs indicate right and left advances in time, respectively). Each stimulus was 400-ms long, including 100-ms raised-cosine onset and offset ramps. The raised cosine ramps at the onset and offset of the stimulus were synchronized between the two ears. Signal and non-signal intervals were separated by a 200-ms silent gap. The listeners were required to indicate the direction of the ITD change between the two intervals on the basis of the laterality of sound images. In each tracking session, ΔITD started from 100 to 400 μs, for low- or high-frequency stimuli, respectively. For the first four turnpoints, ΔITD was increased or decreased by a factor of 10^0.2^ after one incorrect response or two consecutive incorrect responses, and for the following eight turnpoints, the factor was reduced to 10^0.05^. The threshold for the session was computed as the geometric mean of the ΔITD at the last eight turnpoints.

***ILD task***. In a 2I-2AFC trial, stimuli in the two intervals had ILDs of +ΔILD/2 and −ΔILD/2 dB, respectively (positive and negative ILDs indicate higher and lower levels in the right ear, respectively). Each stimulus was 400-ms long, including 20-ms raised-cosine onset and offset ramps. The listeners were required to indicate the direction of the ILD change between the two intervals on the basis of the laterality of sound images. In each tracking session, ΔILD started from 2.5 dB. For the first four turnpoints, ΔILD was increased or decreased by 0.5 dB after one incorrect response or two consecutive incorrect responses, and for the following eight turnpoints, the step size was reduced to 0.25 dB. The threshold for the session was computed as the mean of the ΔILD at the last eight turnpoints. Other details were the same as in the ITD task.

***Time task***. For the low-frequency stimulus, the listeners were required to detect a common upward frequency shift (Δ*f* Hz) imposed on the individual components of the SSMC with the spectral envelope remaining unchanged. The stimulus parameters and measurement methods for a detection threshold for the frequency shift was in accordance with the “TFS1” test developed by Moore and Sek (2009). It has been reported that such a shift in component frequencies is accompanied with shift in pitch (De Boer, 1956; Schouten et al., 1962; Moore and Moore, 2003). This pitch change was considered to be largely the result of changes in the TFS, since individual frequency components were only intermediately resolved in the auditory periphery (Moore and Moore, 2003) and frequency spacing (corresponding to the periodicity of the envelope) was unchanged. In addition, frequency shifts around a typical threshold value are expected to alter the peripheral excitation pattern by a negligible amount (Moore and Sek, 2009). Therefore, we adopted this task for evaluating the efficiency of neural phase locking to TFS. It should be noted that the pitch of the frequency-shifted SSMC is often ambiguous and listeners could base their judgments not on pitch shifts but on inharmonicity when conducting the tasks (De Boer, 1956; Schouten et al., 1962), and that it was not our intention to use this task for evaluating the pitch mechanism. The “signal” and “non-signal” intervals in the 2I-2AFC method contained RSRS and RRRR sequences, respectively, where R indicates a harmonic complex (i.e., original SSMC) as the reference and S indicates a frequency-shifted SSMC. The listener was required to indicate the signal interval (RSRS).

To assess the peripheral efficiencies of nerural phase locking to stimulus envelope at a high frequency, we adopted a task to measure discriminability of the transposed stimuli with modulation frequencies of 125 Hz and 125 + Δ*f*_*m*_ Hz, referred to as R and S, respectively. Similarly to the low-frequency stimulus, the listener was required to indicate the signal interval (RSRS) as opposed to the non-signal interval (RRRR). When preforming this task, the listeners could base their judgments on changes in pitch associated with the modulation frequency, although the pitch sensation of the transposed stimulus is generally weak and ambiguous (Oxenham et al., 2004).

Commonly for the low- and high-frequency stimuli, an R or S tone had a duration of 100 ms, including 20-ms raised-cosine ramps. There were 100-ms silent intervals between the tones within a sequence in one interval, and there was a 300-ms silent gap between the intervals. In one session of adaptive tracking, Δ*f* or Δ*f*_*m*_ was increased or decreased by a factor of 2^0.5^ after one incorrect response and after two consecutive correct responses, respectively, for the first four turnpoints. The factor was reduced to 2^0.25^ for the following eight turnpoints. The geometric mean of Δ*f* or Δ*f*_*m*_ was computed across the last eight turnpoints, which represented the threshold for the session.

The maximum frequency shift, Δ*f*, was limited to 50 Hz (i.e., 0.5 × *F*_0_ Hz) in the adaptive tracking for the low-frequency stimulus. For three listeners, the adaptive tracking failed to converge within the maximum Δ*f* limit (50 Hz) for at least one session. For those listeners, their performance was evaluated by the method of constant stimuli, instead of the adaptive method. Subjects were given the same instructions as for the adaptive procedure. A session consisted of 20 trials, and subjects completed five sessions. The Δ*f* was fixed at the maximum value, 50 Hz. The proportion of correct responses was derived from the pooled responses across 10–12 sessions, and converted to *d*' (Hacker and Ratcliff, 1979). To make the results comparable to the measures obtained by the adaptive method, the threshold was derived on the assumption that *d*' is proportional to the frequency shift (Hopkins and Moore, 2007) and that the adaptive procedure tracked the 70.7% correct point on the psychometric function, which corresponds to a *d*' of 0.77 with a 2AFC task. This method sometimes yielded values of the threshold greater than the maximum Δ*f* limit of 50 Hz. Although such large values of thresholds could not be measured empirically, they could be taken as indicators of the listeners' performance.

***Level task***. In a 2I-2AFC trial, the listeners were required to indicate an interval containing a 400-ms-long SSMC or a transposed stimulus whose central 200-ms portion (including 20-ms raised-cosine ramps) was incremented in level by Δ*L* dB, while the other non-signal interval contained an original SSMC or a transposed stimulus. In one session of adaptive tracking, Δ*L* started with 6 dB and was increased or decreased by a factor of 2.68 after one incorrect response and after two consecutive correct responses, respectively, for the first four turnpoints. The factor was reduced to 1.67 for the following eight turnpoints. The geometric mean of Δ*L* was computed across the last eight turnpoints, which represented the threshold for the session.

## Results

**Figure 1:**
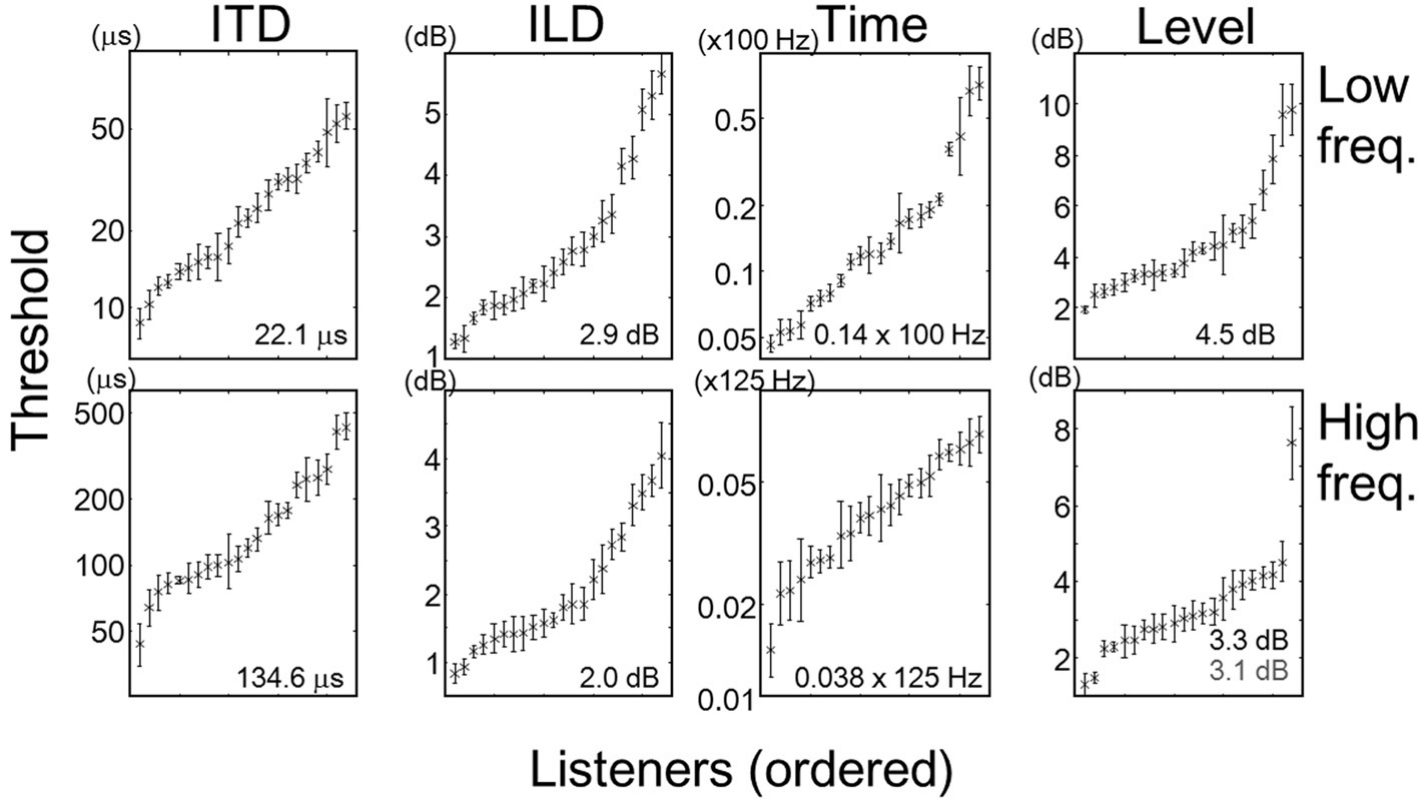
**Means and standard errors of individual listeners' thresholds, expressed by the crosses and error bars, respectively.** Each panel represents one task, and each set of cross and error bar represents one listener. Within each panel, listeners are sorted according to the mean threshold. Note that for the ITD and Time tasks, the thresholds have been log-transformed. Numbers in the panel indicate the mean across the listeners. In the panel for the high frequency Level task, the number in gray indicates the mean calculated excluding listener 10 (rightmost data).

Threshold data for individual tasks and listeners are summarized in Figure 1. Each symbol and error bar represents the mean and standard error of thresholds of one listener obtained from multiple sessions. Within each task, the listeners are sorted according to the mean threshold. It should be noted that for the ITD and Time tasks, the means and standard errors are represented on a logarithmic scale. Note also that the thresholds for the low- and high-frequency Time tasks are expressed as fractions to *F*_0_ (100 Hz) and modulation rate (125 Hz), respectively. The number in each panel indicates the average across the listeners. One listener (listener number: 10) exhibited an extremely large threshold in the high-frequency Level task (see the rightmost data in the corresponding panel). In the following sections, we report the results of correlation and multiple-regression analyses with and without this listener when they are related to the high-frequency Level task.

**Figure 2:**
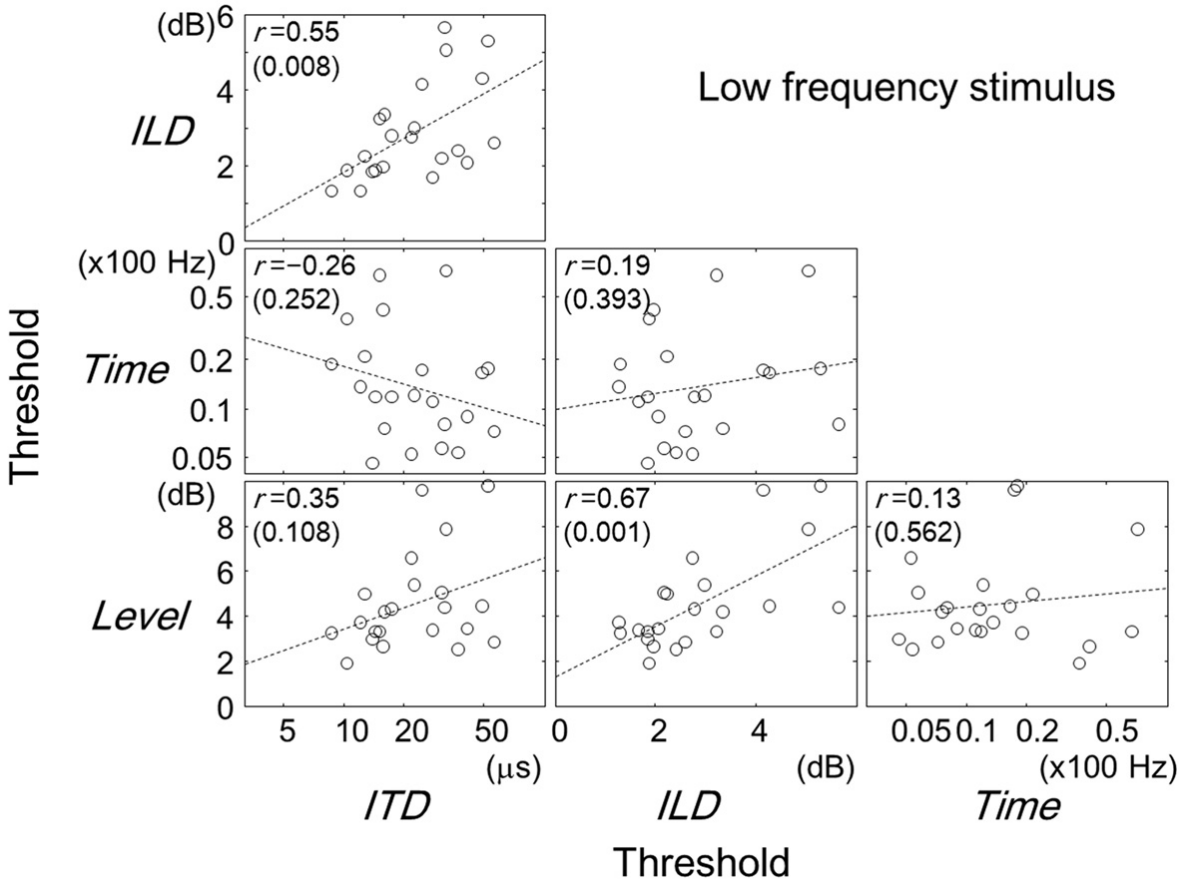
**Comparisons of thresholds between tasks for the low-frequency stimulus.** Each panel represents one combination of tasks as labeled. Each symbol represents one listener. The broken lines are best-fit straight lines to the data. The Pearson correlation coefficients are shown with their *p*-values in parentheses. Note that for the ITD and Time tasks, the thresholds have been log-transformed. A similar figure has appeared elsewhere (Furukawa et al., 2013).

**Figure 3:**
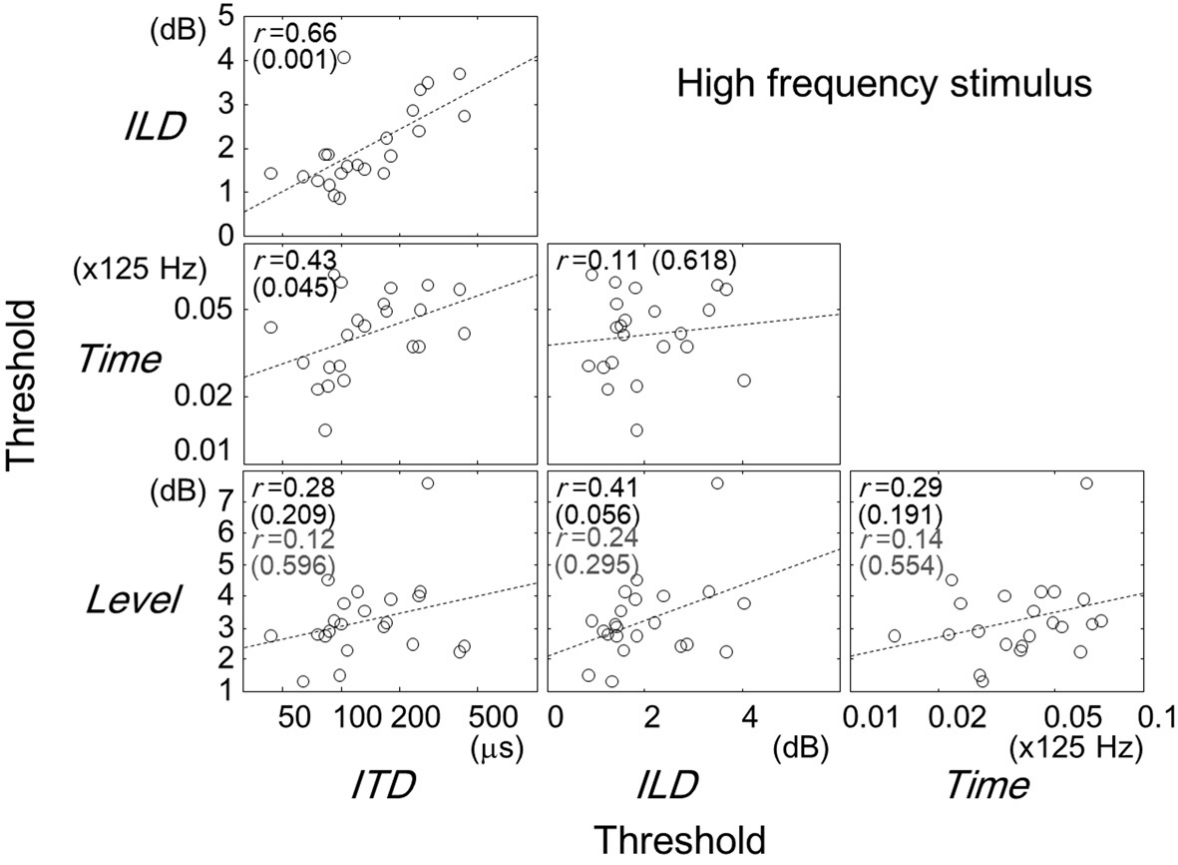
**Same as Figure 2 but for the high-frequency stimulus.** The correlation coefficients and *p*-values in gray indicates values when listener 10 was excluded.

**Figure 4:**
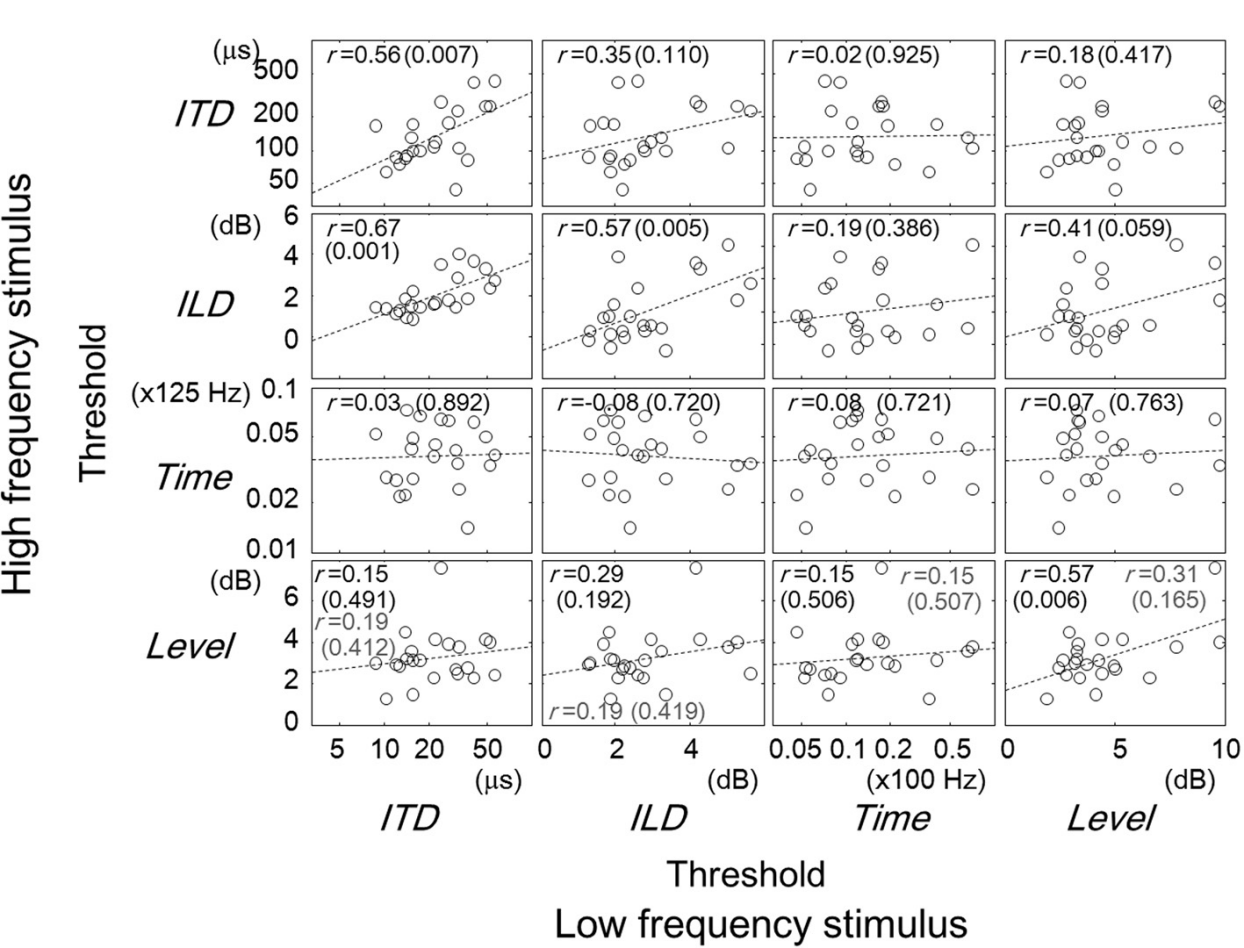
**Comparisons of thresholds between tasks for different frequency regions.** The panels are arranged so that the horizontal and vertical axes represent the data for the low- and high- frequency stimuli, respectively. Other conventions are the same as in Figures 2, 3.

Figures 2–4 show scatter plots comparing individual listeners' thresholds between pairs of tasks. Each panel in the figures shows the data for one combination of tasks, representing 22 listeners with data points. For the Time and ITD tasks, we converted the thresholds to a logarithmic scale when plotting the data and computing the Pearson correlation coefficients.

### Low-frequency stimulus

Focusing on the results for the low-frequency stimulus (Figure 2), one can see statistically significant positive correlations for pairs of ITD and ILD tasks (*r* = 0.55; *p* = 0.008) and of ILD and Level tasks (*r* = 0.67; *p* = 0.001). The pair of Time and ITD tasks showed a weak negative correlation (*r* = −0.26), which was, however, not statistically significant (*p* = 0.252).

We used a multiple linear regression analysis to further explore the factors that might account for inter-individual variability in the lateralization tasks, which might not be revealed by the single correlation analysis. For a given lateralization task of interest (“target task”; i.e., ITD or ILD task), we regarded the threshold for that task as the dependent variable and the thresholds for the remaining three tasks as the explanatory variables. A significant partial correlation of an explanatory task would suggest that the performance of that explanatory task is a good predictor of the performance of the target task. The size of partial correlation coefficient for each explanatory variable could be interpreted as indicating the size of the effect of the variable (or of mechanisms behind the variable) on the performance of the target task, given the values of the other variables are fixed.

The regression analyses were conducted on the threshold data which had been transformed to *z* scores (i.e., having a mean of 0 and a standard deviation of 1), for individual tasks. Estimated values of partial correlation coefficients are summarized in Table 1, along with *p* values indicating whether the coefficient was significantly different from zero. For the ITD task as the target, the partial correlation coefficient was significant for the ILD task (*p* = 0.015). As for the ILD task as the target, the coefficients for the ITD and Level tasks were significant (*p* = 0.015 and 0.008, respectively).

**Table 1:**
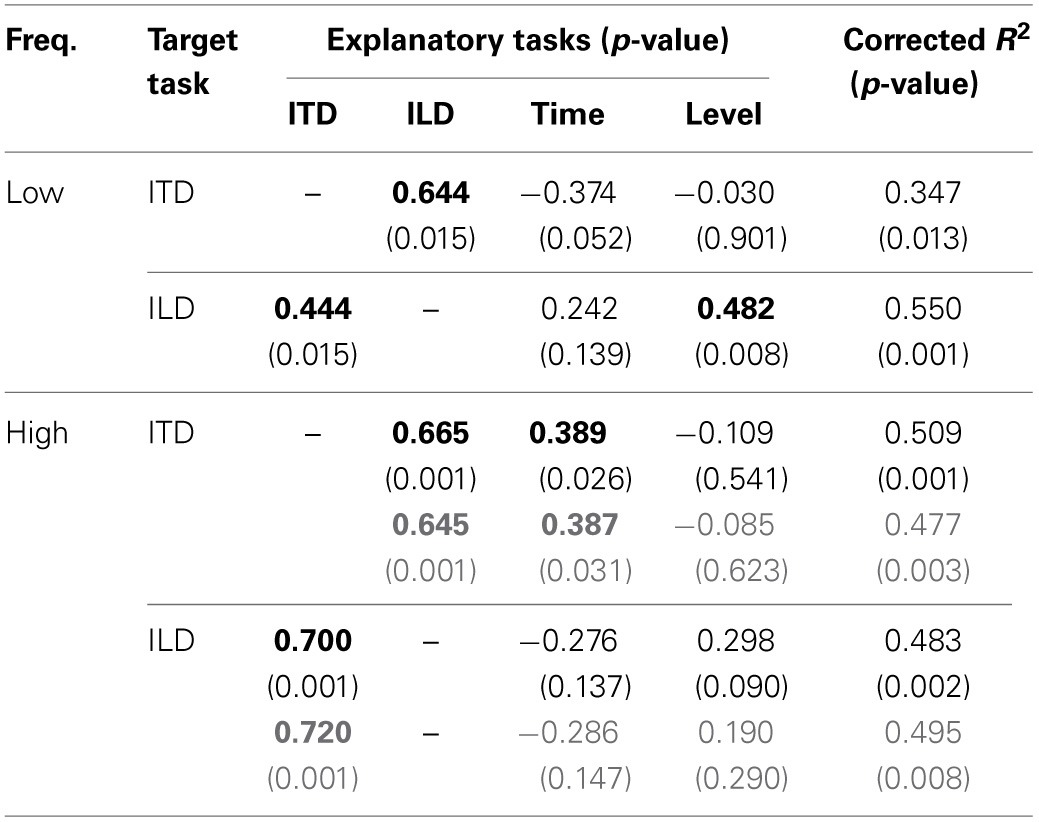
**Summary of multiple regression analyses for low- and high-frequency stimuli**.

Partial correlation coefficients and the p-values are shown for individual explanatory tasks. Note that the analyses were conducted on the z scores of the threshold data. The bold characters indicate statistically significant correlation (p < 0.05). The rightmost column shows the multiple coefficients of determination (adjusted for degrees of freedom) and their p-values. For the high frequency stimulus, the results obtained when listener 10 was excluded are also shown in gray.

### High-frequency stimulus

Comparisons between the thresholds of the task types for the high-frequency stimulus are represented in Figure 3. Significant correlation were found for pairs of the ITD and ILD tasks (*r* = 0.66, *p* = 0.001), and of the ITD and Time tasks (*r* = 0.43, *p* = 0.045). The correlation of the ILD and Level tasks was not significant (*r* = 0.41, *p* = 0.056; *r* = 0.24, *p* = 0.295, when listener 10 was excluded).

The results of the multiple linear regression analysis are shown in Table 1. Consistent with the results of the single correlation analysis, the partial correlation coefficients of the ILD and Time tasks were significant when the ITD task was the target (*p* = 0.001 and 0.026, respectively). The coefficint of the ITD task was significant when the ILD was the target task (*p* = 0.001). Exclusion of listener 10 did not affect the general conclusions of the analysis.

### Across-frequency comparisons

The correlation of task performance across frequencies can be examined in Figure 3. When comparing the thresholds for the same task type, one can see that the correlations were significant for all the tasks except the Time task (*r* = 0.56, *p* = 0.007 for ITD; *r* = 0.57, *p* = 0.005 for ILD; *r* = 0.08, *p* = 0.721 for Time; *r* = 0.57, *p* = 0.006 for Level). The correlation for the Level tasks, however, became non-significant when listener 10 was excluded (*r* = 0.31, *p* = 0.165). A significant correlation for different task types was found in the combination of low-frequency ITD and high-frequency ILD tasks (*r* = 0.67, *p* = 0.001). Significant correlations across frequency regions imply an across-frequency factor that determined the performance of a given task for a frequency region.

Here again, we conducted a multiple linear regression analysis using thresholds (in *z* scores) of all the combinations of task and stimulus as independent variables. In this analysis, we were specifically interested in the extent to which the performance of one lateralization task could be accounted for by the performances of other tasks, whether the stimuli were in the same or remote frequency regions, and in identifying tasks where the performance could predict the performance of the target. We used Akaike's information criterion (AIC) as a basis for selecting most effective combination of variables for the regression while avoiding overfitting (Burnham and Anderson, 2002, p. 63). The AIC values were obtained individually for models with all possible combinations of explanatory variables using the LinearModel.fit function of MATLAB. The combination of variables exhibiting the lowest AIC was employed for constructing the linear model. The results of the analysis are summarized in Table 2.

**Table 2:**
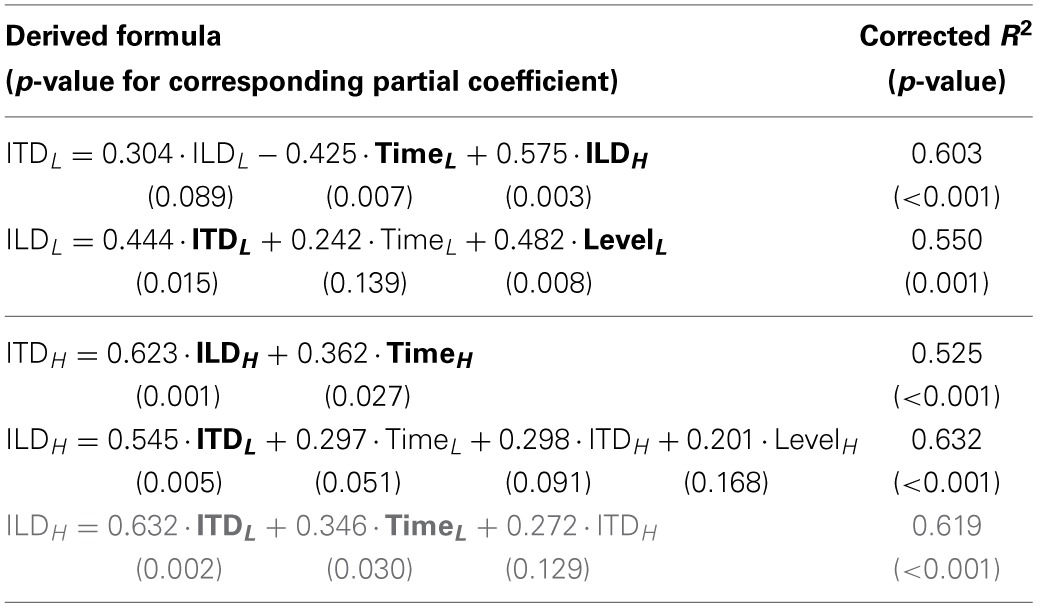
**Summary of multiple regression analyses on all tasks**.

For each target task, explanatory variables (or tasks) were selected based on the AIC (see text). Each symbol (e.g., ITD_L_) represents the threshold (in z score) of the corresponding task and stimulus (subscripts of L and H represent low- and high-frequency stimuli, respectively). Partial correlation coefficients and p-values are shown for individual explanatory tasks. The bold characters indicate statistically significant coefficients (p < 0.05). The rightmost column shows the multiple coefficients of determination (adjusted for degrees of freedom) and their p-values. For the high frequency ILD task (ILD_H_), a different result of variable selection was obtained when listener 10 was excluded (indicated in gray).

The linear model could account for a relatively large fraction of the variance of the threshold in a target task (*R*^2^ ranged between 0.525 and 0.632). In addition, the results of the variable selection were generally in accordance with the findings described earlier: For a given target task and stimulus frequency, the other lateralization task at the same frequency was selected as an explanatory variable (e.g., for the target of the low-frequency ITD task, the low-frequency ILD task was selected), although the coefficients were not always significantly different from zero. It was also confirmed that for target tasks of ITD and ILD tasks, selected explanatory variables included the Time and Level tasks, respectively. The partial correlation coefficient for the low-frequency ITD task was significant and negative for the target task of low-frequency Time (−0.425; *p* = 0.007). Exclusion of listener 10 affected the result for the target task of high-frequency ILD: the high-frequency Level task was no more selected, and the partial correlation coefficient for the low-frequency Time task became significant (0.346; *p* = 0.030).

### Principal component analysis

**Figure 5:**
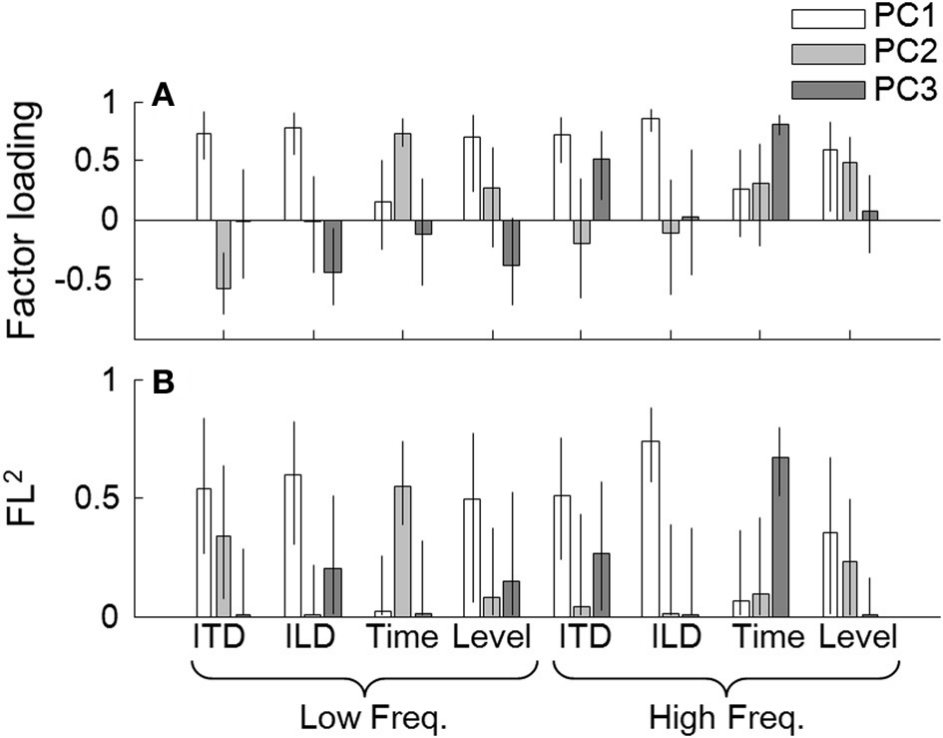
**(A)** Factor loadings on the individual tasks, derived by the principal component analysis. The loads for the three components (i.e., PC1, PC2, and PC3) are represented by the three bars as indicated in the key. The error bars indicate 95% confidence intervals of the loads estimated by the bootstrap method. **(B)** Squared factor loadings (indicating the proportion to the total variance for the task accounted for by that component). Other conventions are the same as in **(A)**.

So far, we have examined associations across tasks through single correlation and the multiple linear regression analyses. Interpretations of the coefficients, however, are often difficult when there are marked correlations among the explanatory variables, which was often the case in the present study. It was possible that the performance of the tasks evaluated in the present study could be explained by one or more common underlying factors. To examine this, we conducted a principal component analysis (PCA) on vectors of the eight tasks obtained from the 22 listeners. Before running the analysis, the threshold data were transformed to a logarithmic scale (for the Time and ITD tasks only) and then to *z*-scores (all the measures). The results indicated that the data could be accounted for well by the first three principal components (PCs; from PC1 to PC3), which had eigenvalues of 3.33, 1.34, and 1.30, respectively. These three PCs accounted for 74.6% of total variance. The factor loadings (FLs) of the three PCs (indicated by gray-scaled bars) and their squared values (FL^2^s) are shown in Figures 5A,B, respectively. The FL^2^ for a given task by a given PC indicates the proportion to the total variance for the task accounted for by that component.

For all four lateralization tasks, the FL^2^ values by PC1 were above 0.5. PC1 had positive loads on all the tasks (Figure 5B), implying that PC1 reflects the general ability of the listeners to conduct psychophysical tasks. Note, however, that the loads on the low- and high-frequency Time tasks were relatively small. Also, there were marked contributions of PC2 and PC3, depending on the task. For the low-frequency ITD task, PC2 could account for more than 30% of the variance. An examination of FLs revealed that PC2 was associated predominantly with the low-frequency Time task (Figure 5B), and the FLs on the low-frequency ITD and Time tasks had opposite signs (Figure 5A). This implies that PC2 reflects a factor that had opposing effects on the Pitch and ITD tasks at low frequency. PC3 had appreciable contributions to low-frequency ILD and high-frequency ITD tasks. PC3 was associated with the high-frequency Pitch task, which had the same sign as the FL on the high-frequency ITD task. To a lesser degree, PC3 also showed some association with the low-frequency Level task, which had the same sign as the FL on the low-frequency ILD task. Exclusion of listener 10 did not alter the general conclusions of the analysis.

## Discussion

The major findings of the present study were: positive correlations between the performance of pairs of lateralization tasks (i.e., ITD and ILD tasks) both within and across stimulus frequencies; a negative correlation for the low-frequency ITD and the Time tasks, revealed by the multiple-regression analysis; a positive correlation for the high-frequency ITD and the Time tasks; and a positive correlation for the low-frequency ILD and the Level tasks.

The mean thresholds obtained in the present study were generally at the same levels of those obtained by earlier comparative studies: ITD: Bernstein and Trahiotis (2002), Furukawa (2008); ILD: Grantham (1984), Furukawa (2008); Time: Plack and Carlyon (1995), Moore and Sek (2009); Level: Moore et al. (1997). Thresholds in the ITD task for the high frequency stimulus were greater than those for the low frequency stimulus by an order of magnitude. This quantitative difference is likely due to the difference in the tone and modulator frequencies and does not immediately indicate mechanistic difference between the frequencies: Typical threshold ITD for the 125-Hz tone, which is considered to be equivalent to the present transposed stimulus in terms of the peripheral phase locking, is comparable to the threshold for the transposed stimulus (see Bernstein and Trahiotis, 2002).

The significant positive correlations generally found between the performance of pairs of lateralization tasks indicate that some degree of inter-individual variation of performance could be accounted for by a common factor or mechanism that underlies lateralization based on both ITDs and ILDs over frequency regions. This notion is supported further by the fact that PC1 found in the PCA had large contributions to all the lateralization tasks. Furukawa (2008) found that the degree of ITD and ILD interaction is greater at high frequency than at low frequency, indicating that the dominance of a common mechanism depends on stimulus frequency or that different mechanisms for ITD and ILD processing are involved for low- and high-frequency stimulus. The present analyses regarding ITD-ILD relations, however, provided no indication of frequency-dependent processes for ITDs and ILDs: The correlation coefficients for the ITD and ILD pairs were not significantly different between low- and high-frequency stimuli (*p* = 0.581; *t*-test after the Fisher transformation of the correlation coefficients). One candidate for such a mechanism is a binaural mechanism that can process both ITDs and ILDs and can operate across frequency regions. Unfortunately, the present study cannot rule out another candidate, which is a non-sensory, higher-order factor related to the experimental procedure. It is possible that the inter-listener variability in the lateralization performance reflected predominantly the difference in procedure-specific skills. It was common across all the lateralization tasks that the listener had to identify the direction in which (toward left or right) intracranial images of two successive stimulus intervals changed. In the other tasks, on the other hand, the listener was asked to choose the interval that would contain changes in stimulus attributes.

The performance of the ITD task for the high frequency stimulus showed a significant positive correlation with that of the Time task. The following multiple-regression analyses also indicated a significant contribution of the high-frequency pitch task performance to account for the individual variability of the ITD performance. This tendency was captured in PC3 revealed by the PCA, suggesting that this positive correlation reflects a factor that is independent of another non-task-specific factor that determines the listener's overall psychophysical performance (expressed as PC1) or a factor that reflects the relationship of ITD and Time tasks (expressed as PC2; described later). This finding supports our initial hypothesis that the efficiency of neural phase locking to envelope of high frequency stimulus has a significant contribution to ITD-based lateralization performance.

For the low frequency stimulus, however, we failed to observe a positive correlation in the ITD and Time task pairs for the low frequency stimulus. This failure may be attributable to difference in the order of magnitude required for the two tasks: In the low-frequency Time task, a typical threshold of 10-Hz frequency shift of our SSMC stimulus is considered to correspond to difference in peak-to-peak time of TFS by about 100 μs (see Moore, 2012 pp. 220-223), which is an order of magnitude greater than a typical ITD threshold of 20 μs. For the high frequency, on the contrary, a typical threshold Δ*f*_*m*_ of 4 Hz corresponds to change in the peak-to-peak interval of the modulation by about 250 μs, which falls in the range of ITD thresholds.

It is interesting that the across-frequency multiple-regression analysis with a variable selection procedure (Table 2) revealed that the low-frequency Time-task performance was a significant predictor of the low-frequency ITD-task performance, and it had a *negative* contribution. This negative relationship was observed also as the opposite signs of the FLs for the two tasks in PC2, an independent factor (Figure 5). This negative relationship not only was unexpected on the basis of our initial hypothesis but also appears to contradict to earlier reports on hearing-impaired or aged listeners (Strelcyk and Dau, 2009; Hopkins and Moore, 2011). This discrepancy among studies could be explained by postulating two factors that determine the listener's sensitivities to ITDs and the TFS: One factor, associated with the negative correlation, is dominant for normal-hearing listeners. As hearing impairment progresses, the other factor would dominate, resulting in a positive correlation in a population of normal- and hearing-impaired listeners.

One might be concerned about the listener's use of the excitation-pattern or spectral cue as a confounding factor for this negative relationship. Although the change in the excitation level for a typical threshold value (around Δ*f*/*F*_0_ = 0.1) was expected to be negligible (Moore and Sek, 2009), listeners who exhibited relatively high threshold might rely on the excitation pattern cue, which was usable for frequency shifts near their thresholds. Those listeners might be simply insensitive to the TFS information or might have adapted to placing more weights on the spectral cue than on the temporal cue in pitch judgments through their long-term experience (McLachlan et al., 2013). However, it is difficult to explain the negative correlation in terms of the use of the excitation-pattern cue: Listeners with general insensitivities to TFS would be expected to be insensitive to ITD also, leading to a positive correlation. We cannot think of obvious association between larger weighting on the place over the temporal cues and better (or poorer) performance in the ITD task.

One explanation for the puzzling negative correlation is that the listeners could use two types of ITD cues when conducting the ITD task, namely, envelope and TFS-based ITDs (since ITDs were imposed on both of those properties), and the performance depended on the relative weights placed on the two cues by individual listeners. It is possible that the envelope ITD of our stimulus was more reliably coded in the auditory system than the TFS-based ITD was. In the Time task, on the other hand, the TFS information could be the main cue for the judgments (although other types of information, such as distortion products by cochlear non-linearity and the excitation pattern, are also arguably potential cues, Oxenham et al., 2009; Micheyl et al., 2010), while the temporal envelope of the stimulus provided no useful cue, since it always had the same repetition rate (100 Hz). Therefore, a listener who places a greater weight on the envelope cue would tend to exhibit better and poor performance in the ITD and Pitch tasks, respectively. It should be noted that this explanation assumes that individual listeners applied more or less the same relative weights on the envelope and TFS invariantly in the Time and ITD tasks.

As for the relationship between the ILD and Level tasks, a significant positive correlation for the low-frequency stimulus supports our initial hypothesis that, at least for the low frequency stimulus, the inter-individual variability of ILD performance reflects the difference in the efficiency of intensity coding at a processing stage earlier than binaural interaction. One might be concerned that the listeners in the ILD task based their judgments primarily on the change of stimulus level within a single ear, and thus the ILD task measured essentially monaural sensitivity to level change. However, this is not likely, as supported by the suggestion of Bernstein (2004) that the listener's judgment is likely to be based on changes in the position of an intracranial image, not on the monaural cues.

## Author contributions

Author Atsushi Ochi designed and conducted the experiments, analyzed the data, and prepared the manuscript. Tatsuya Yamasoba designed the experiments. Shigeto Furukawa conceived and designed the experiments, analyzed the data, and prepared the manuscript.

### Conflict of interest statement

The authors declare that the research was conducted in the absence of any commercial or financial relationships that could be construed as a potential conflict of interest.
